# Ischemia time impacts on respiratory chain functions and Ca^2+^-handling of cardiac subsarcolemmal mitochondria subjected to ischemia reperfusion injury

**DOI:** 10.1186/s13019-019-0911-1

**Published:** 2019-05-14

**Authors:** Marcus Leistner, Stefanie Sommer, Peer Kanofsky, Rainer Leyh, Sebastian-Patrick Sommer

**Affiliations:** 1 Department of Thoracic, Cardiac and Vascular Surgery, University Medical Center Goettingen, Goettingen, Germany; 2 Department of Thoracic and Cardiovascular Surgery, Herz- und Diabeteszentrum Nordrheinwestfalen (HDZ-NRW), Bad Oeynhausen, Germany; 3 Institute for Laboratory and Transfusion Medicine, Herz- und Diabeteszentrum Nordrheinwestfalen (HDZ-NRW), Bad Oeynhausen, Germany; 4 Department of Cardiothoracic and Thoracic Vascular Surgery, University Hospital Wuerzburg, Wuerzburg, Germany

**Keywords:** Subsarcolemmal mitochondria, Ischemia reperfusion injury, Ischemia time

## Abstract

**Background:**

Mitochondrial impairment can result from myocardial ischemia reperfusion injury (IR). Despite cardioplegic arrest, IR-associated cardiodepression is a major problem in heart surgery. We determined the effect of increasing ischemia time on the respiratory chain (RC) function, the inner membrane polarization and Ca^2+^ homeostasis of rat cardiac subsarcolemmal mitochondria (SSM).

**Methods:**

Wistar rat hearts were divided into 4 groups of stop-flow induced warm global IR using a pressure-controlled Langendorff system: 0, 15, 30 and 40 min of ischemia with 30 min of reperfusion, respectively. Myocardial contractility was determined from left ventricular pressure records (dP/dt, dPmax) with an intraventricular balloon. Following reperfusion, SSM were isolated and analyzed regarding electron transport chain (ETC) coupling by polarography (Clark-Type electrode), membrane polarization (JC1 fluorescence) and Ca^2+^-handling in terms of Ca^2+^-induced swelling and Ca^2+^-uptake/release (Calcium Green-5 N® fluorescence).

**Results:**

LV contractility and systolic pressure during reperfusion were impaired by increasing ischemic times. Ischemia reduced ETC oxygen consumption in IR40/30 compared to IR0/30 at complex I-V (8.1 ± 1.2 vs. 18.2 ± 2.0 nmol/min) and II-IV/V (16.4 ± 2.6/14.8 ± 2.3 vs. 2.3 ± 0.6 nmol/min) in state 3 respiration (*p* < 0.01). Relative membrane potential revealed a distinct hyperpolarization in IR30/30 and IR40/30 (171.5 ± 17.4% and 170.9 ± 13.5%) compared to IR0/30 (*p* < 0.01), wearing off swiftly after CCCP-induced uncoupling. Excess mitochondrial permeability transition pore (mPTP)-gated Ca^2+^-induced swelling was recorded in all groups and was most pronounced in IR40/30. Pyruvate addition for mPTP blocking strongly reduced SSM swelling in IR40/30 (relative AUC, ± pyruvate; IR0/30: 1.00 vs. 0.61, IR15/30: 1.68 vs. 1.00, IR30/30: 1.42 vs. 0.75, IR40/30: 1.97 vs. 0.85; *p* < 0.01). Ca^2+^-uptake remained unaffected by previous IR. Though Ca^2+^-release was delayed for ≥30 min of ischemia (*p* < 0.01), Ca^2+^ retention was highest in IR15/30 (RFU; IR0/30: 6.3 ± 3.6, IR 15/30 42.9 ± 5.0, IR30/30 15.9 ± 3.8, IR40/30 11.5 ± 6.6; *p* ≤ 0.01 for IR15/30 against all other groups).

**Conclusions:**

Ischemia prolongation in IR injury gradually impaired SSM in terms of respiratory chain function and Ca^2+^-homeostasis. Membrane hyperpolarization appears to be responsible for impaired Ca^2+^-cycling and ETC function. Ischemia time should be considered an important factor influencing IR experimental data on subsarcolemmal mitochondria. Periods of warm global ischemia should be minimized during cardiac surgery to avoid excessive damage to SSMs.

## Background

Myocardial dysfunction after prolonged ischemia is one most important reason for delayed recovery after cardiac surgery. Mitochondrial functional impairment has been linked to the underlying postoperative cardiac depression. As ischemia reperfusion (IR) injury negatively impacts on the preservation of the inner mitochondrial membrane (IMM) potential through disruption of the electron transport chain (ETC) coupling, consecutive ATP depletion and intracellular calcium overload following reduced mitochondrial calcium retention capacity determine the loss of cardiac contractility [1].

Cardiac mitochondria have long been recognized to consist of interfibrillar and subsarcolemmal populations [2]. These subgroups show distinct morphological and biochemical differences, e. g. with respect to membrane channel expression and different affection by aging [3, 4]. They also feature typical properties in dealing with pathological stimuli. Specifically, subsarcolemmal mitochondria (SSM) are suspected to be more sensitive towards calcium challenges and IR injury rather than their interfibrillar (IFM) counterparts [5–7]. As ischemic periods exceeding 20 min lastingly compromise the overall mitochondrial function and integritiy [8, 9], we hypothesized that isolated SSM might be particularly affected in a time dependent manner by increasing intervals of ischemia during IR. We sought to comprehensively determine the influence of different durations of IR on calcium homeostasis, electron transport chain coupling and IMM polarization changes specifically in SSM in a Langendorff perfusion model of isolated rat hearts. As intermittent cardioplegic arrest and hypothermic conditions should ameliorate any ischemia-induced effects, we sought to maximize the discriminatory power of our experiments by excluding such confounders.

## Methods

### Langendorff heart preparation and isolation of mitochondria

Animal use was approved by local authorities before commencing experiments. Animals were treated according to the Principles of Laboratory Animal Care and the Guide for the Care of Use of Laboratory Animals prepared by the Institute of Laboratory Animal Resources, National Research Council, and published by the National Academy Press (rev. 1996) as well as in compliance with the European Convention on Animal Care. Unless stated otherwise, chemicals were obtained from Sigma Aldrich GmbH (Munich, Germany).

A total of 60 adult male Wistar rats (220–280 g; Janvier, St. Berthevin Cedex, France) were anesthetized by an isoflurane/oxygen mixture (5/95%), decapitated and hearts were excised. After retrograde aortic cannulation, perfusion was initiated for 2 min with Custodiol® solution in order to prepare for a maximum of 3 min transport time before mounting the heart to a Langendorff apparatus (Föhr Medical Instruments GmbH (FMI), Seeheim, Germany). All hearts were perfused with carbogen-gased (5% CO_2_, 95% O_2_) Krebs-Henseleit bicarbonate (KHB) buffer (118 mM NaCl, 4.7 mM KCl, 1.25 mM CaCl_2_, 1.2 mM MgSO_4_, 1.2 mM KH_2_PO_4_, 25 mM NaHCO_3_, 11 mM glucose) at a constant pressure of 73.6 mmHg. After 5 min of equilibrating coronary perfusion, hearts were allocated to groups of different durations of warm (37 °C) ischemia followed by 30 min of reperfusion (control group = IR0/30, *n* = 30; IR15/30, *n* = 10; IR30/30, n = 10; IR40/30, n = 10). During reperfusion, left ventricular end diastolic pressure (LVEDP) was set to 18 mmHg via antegrade balloon placement in the left ventricle through the exposed mitral valve. The balloon was kept inflated at the designated LVEDP through constant manual adjustment using a Hamilton syringe. Left ventricular pressure (LVP) and left ventricular contractility (LVdp/dtmax) were recorded using LabChart, Version 5 (ADInstruments, Oxford, United Kingdom), throughout the reperfusion phase.

At the end of the experiment, hearts were immersed in iced KHB buffer for isolation of mitochondria. SSM were isolated according to Palmer et al. [2] in a buffer of 180 mM KCl, 10 mM EDTA and 0.5% albumin, pH 7.4. Cardiac tissue was minced, subjected to differential centrifugation and SSM pellets were washed using a buffer of 225 mM mannitol, 75 mM sucrose and 20 mM Tris, pH 7.4. SSM protein concentration was determined from a bicinchoninic assay according to manufacturer’s protocol (BCA Kit, Thermo Fisher Scientific, Schwerte, Germany).

### Analysis of mitochondrial respiratory chain complexes

The five complexes of the respiratory chain (complex I: NADH dehydrogenase; complex II: succinate dehydrogenase; complex III: Q-cytochrome c oxidoreductase; complex IV: cytochrome c oxidase; complex V: ATP synthase) form the molecular basis of cellular respiration. Complexes I-III build up an “electron carrier chain” transferring electrons via redox reactions towards increasingly electronegative acceptor molecules. Complex IV finally passes those electrons to an oxygen molecule. Energy released during the whole process is used to build up a proton gradient over the inner mitochondrial membrane (complexes I, III, IV). This gradient is the substrate of the mitochondrial membrane potential and utilized by complex V for ATP generation.

When substrates of complex I and/or II are added to a suspension of isolated mitochondria, so-called “state 2 respiration” is initiated with low oxygen consumption due to retrograde enzyme inhibition (respiratory control). Upon addition of ADP, ATP generation through complex V breaks down the proton gradient over the IMM, boosting RC activity and oxygen consumption. This is described as “state 3 respiration”.

SSM were transferred to a buffer of 300 mM mannitol, 10 mM KH_2_PO_4_, 10 mM KCl and 5 mM MgCl_2_ at pH 7.2 and 25 °C. Oxygen consumption was quantified using a temperature-controlled Oxytherm® Clarke-type electrode (Hansatech Instruments Ltd., Norfolk, UK) for differentially activated and inhibited respiratory chain complexes.

Analysis of complexes I-V: After equilibration for at least 2 min, malate and pyruvate (10 mM each) were added as substrates. State 2 respiration was measured for 30 s before addition of 0.2 mM adenosine diphosphate (ADP). Consecutively, state 3 respiration was determined for another 30 s.

Analysis of complexes II–V: As much as 300 μg of mitochondrial protein (bicinchinonic assay) were suspended in 300 μl of buffer. After equilibration, complex I was blocked by rotenone (0.2 mM). Mitochondria were energized by succinate (1 M) and state 2 respiration was checked for. State 3 respiration was quantified after addition of ADP (0.2 mM).

### Mitochondrial Ca^2+^ uptake and Ca^2+^-induced swelling

SSM were energized at complex I (2.5 mM pyruvate and malate added to a buffer of 250 mM sucrose, 10 mM MOPS (3-(N-morpholino)propanesulfonic acid) and 0.005 mM EGTA (ethylene glycol-bis (β-aminoethyl ether)-N, N, N′, N′-tetraacetic acid) at pH 7.2, respectively) and incubated with a strictly extramitochondrial calcium probe (0.5 μM Calcium Green-5 N®, Thermo Fisher Scientific, Schwerte, Germany)). Ca^2+^ uptake was triggered by addition of 50 μM Ca^2+^ and light emission at a wavelength of 530 nm could be detected using an Infinite 200 Pro® multimode reader (Tecan Deutschland GmbH, Crailsheim, Germany) following excitation at 500 nm. Ca^2+^ uptake and Ca^2+^ release of SSM were analyzed with regards to I) the slope K of each curve in a nonlinear monophasic regression model and II) the net Ca^2+^ retention span at the end of each experiment.

Mitochondrial permeability transition pore (mPTP) opening was determined from Ca^2+^-induced swelling of SSM either with or without mPTP blockade with 50 mM pyruvate. 50 μM Ca^2+^ was added to SSM suspended in a buffer containing 250 mM sucrose and 5 mM KH_2_PO_4_ (pH 7.2) after energizing respiratory chain complex II with 6 mM succinate and simultaneous rotenone (3 μM) blockade of complex I. Decline of transmitted light absorption at 520 nm (Ultrospec™ 300 spectrophotometer, GE Healthcare, Munich, Germany) indicated SSM swelling.

### Degradation of mitochondrial membrane potential

SSM were suspended in a buffer of 250 mM sucrose, 20 mM Hepes (4-(2-hydroxyethyl)-1-piperazineethanesulfonic acid), 12.5 mM KH_2_PO_4_ and 10 mM MgCl_2_ at pH 7.2.

Complex I was blocked by adding 2 μM rotenone before labelling SSM with 400 nM JC1 (5,50,6,60-tetrachloro-1,10,3,30-tetraethylbenzimidazolylcarbocyanine iodide, Enzo Life Sciences GmbH, Lörrach, Germany). JC1 is a positively charged, membrane permeant dye with potential-dependent aggregation. The process is accompanied by a fluorescence shift from 529 to 590 nm. J-aggregates accumulated within the mitochondrial matrix owing to inwardly negative membrane potential (ΔΨm) build-up following complex II activation with 5 mM succinate. Degradation of ΔΨm was analyzed from a shift in the fluorescence ratio of J-aggregates (orange, 590 nm) to JC1 (green, 525 nm) after addition of an uncoupling agent (CCCP, carbonyl cyanide m-chlorophenyl hydrazine) and detected with an Infinite 200 Pro® multimode reader.

### Statistical analysis

Data were recorded and analyzed using Microsoft Excel 2011 and Graphpad Prism for Mac OS X 6.0e, respectively. Results are plotted as means ± standard error of the mean (SEM). Two-way ANOVA with Tukey’s multiple comparisons test was used for analysis of continuous data. One-way ANOVA with Fisher’s LSD was applied for analysis of grouped nonlinear curve parameters. A *p*-value of <.05 was considered statistically significant.

## Results

### Cardiodynamics

**Fig. 1:**
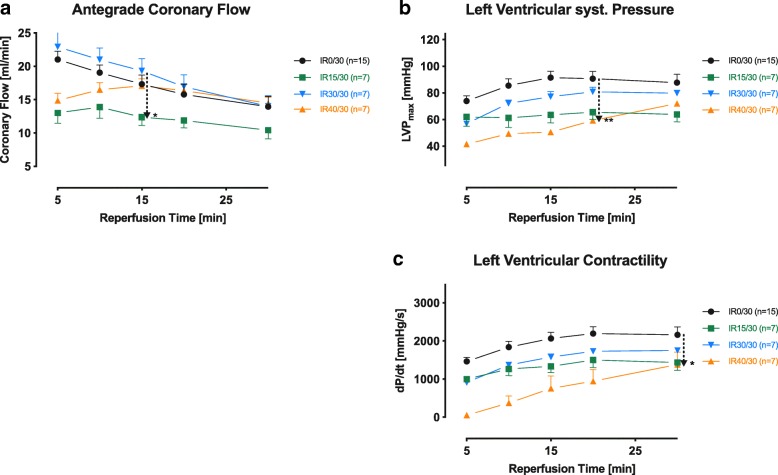
Cardiodynamics. Cardiodynamics are expressed by coronary flow (**a**), left ventricular systolic pressure (**b**) and left ventricular contractility (**c**). Arrows indicate the last pair of group wise significantly different observations during reperfusion. Values are presented as means ± standard error of the mean; *p ≤ 0.05, **p ≤ 0.01

Coronary flow, left ventricular (LV) maximum pressure development and peak contractility were recorded at 5 (pseudo-baseline), 10, 15, 20 and 30 min after the onset of Langendorff retrograde aortic perfusion (Fig. 1). Coronary flow was fairly overshooting, probably due to the immediately preceding application of HTK cardioplegic solution. Our measurements produced somewhat contradictory results: Nearly all initial differences between setups were levelled due to an overall decrease in coronary perfusion over time. However, a significant difference could be detected for up to 15 min between IR15/30 and IR30/30 (*p* = 0.05) caused by a disproportionate decrease at IR15/30 and increase at IR30/30 at the beginning of the recordings. LV maximum systolic pressure and peak contractility of IR40/30 showed a lasting depression until 20 and 30 min of reperfusion compared to IR0/30 (*p* < 0.01 and *p* = 0.02, respectively). More so, depression of LV contractility of IR40/30 was underlined by a significant difference to IR15/30 and IR30/30 during the initial 10 min (*p* = 0.03 and *p* = 0.01, respectively).

### Mitochondrial respiratory chain function

**Fig. 2:**
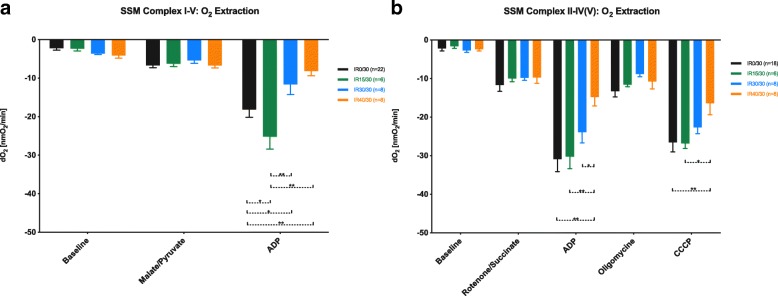
SSM respiratory chain function. Analysis of SSM respiratory chain function by O_2_-extraction from medium at complex I-V (**a**) and II-IV/V (**b**). Bars represent means ± standard error of the mean; *p ≤ 0.05, **p ≤ 0.01. SSM: subsarcolemmal mitochondria

For the analysis of respiratory chain (RC) complexes I-V, SSM were energized exclusively at complex I with pyruvate and malate. State 3 respiration was initiated by adenosine diphosphate (ADP). While prolonged ischemia in IR30/30 and IR40/30 induced a significant reduction of O_2_ consumption compared to IR0/30 (*p* < 0.01 for both), IR15/30 showed an overshooting oxygen demand (*p* = 0.01, Fig. 2a).

The latter could not be detected analyzing state 3 respiration in complexes II-IV/V. SSM were blocked at complex I with rotenone and energized at complex II with succinate followed by addition of ADP. Unlike IR40/30 (*p* < 0.01), neither IR15/30 nor IR30/30 were affected by previous ischemia. After inhibition of adenosine triphosphate (ATP)-synthase with oligomycine and CCCP-induced RC uncoupling (so called “state 3 uncoupled”), a significant effect of IR40/30 on the reduction of O_2_ consumption in comparison to IR0/30 (*p* = 0.01) and IR15/30 (*p* = 0.03) remained detectable (Fig. 2b).

### Analysis of inner mitochondrial membrane potential

**Fig. 3:**
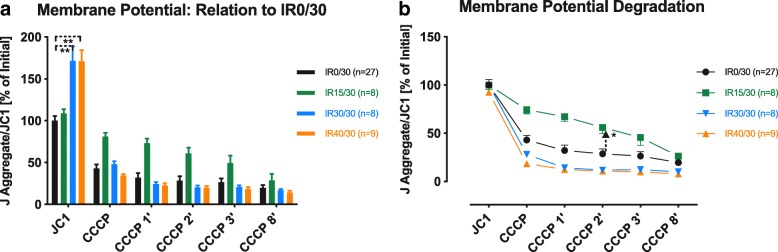
Degradation of mitochondrial membrane potential. Depolarization of ΔΨm due to CCCP-induced uncoupling of complex V. **a** Bars are normalized on IR0/30 to account for ischemia-driven relative changes of ΔΨm. **b** Points are normalized to their individual baseline. Arrow indicates the last pair of significantly different observations during reperfusion. Values are expressed as means ± standard error of the mean; *p ≤ 0.05, **p ≤ 0.01. ΔΨm: inner mitochondrial membrane potential; CCCP: carbonyl cyanide m-chlorophenyl hydrazine; JC1/J-aggregate: 5,50,6,60-tetrachloro-1,10,3,30-tetraethylbenzimidazolylcarbocyanine iodide

Fluorescent J-aggregate formation was recorded after isolated activation of RC at complex II. Figure 3a shows a distinct hyperpolarization of ΔΨm for IR30/30 and IR40/30 at baseline (*p* < 0.01 each).

Following CCCP-induced uncoupling of ΔΨm, normalized IMM potential was stabilized in IR15/30 during the first 2 min of recording (*p* < 0.01). In contrast, neither IR30/30 nor IR40/30 differed significantly from IR0/30 after uncoupling (Fig. 3b).

### Mitochondrial calcium handling

**Fig. 4:**
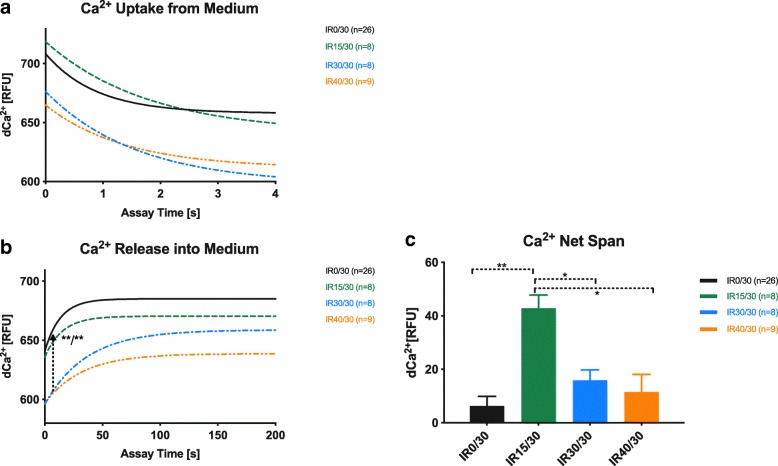
Calcium uptake and release of SSM. SSM calcium handling regarding Ca^2+^ uptake (**a**), liberation (**b**) and net retention (**c**). A and B show fitted nonlinear regression curves, arrow (**b**) indicates significant slope differences of IR30/30 and IR40/30 vs. IR0/30, respectively. Bars in C represent means ± standard error of the mean; *p ≤ 0.05, **p ≤ 0.01. SSM: subsarcolemmal mitochondria; RFU: relative fluorescence units

Ca^2+^ uptake and Ca^2+^ release of SSM are depicted in Fig. 4. Ca^2+^ influx velocity was not significantly affected by the length of ischemic exposure. Compared to IR0/30, slower Ca^2+^ liberation was favored by longer intervals of previous ischemia (K_[IR0/30]_ = 0.07 ± 0.003, K_[IR30/30]_ = 0.02 ± 0.0004, K_[IR40/30]_ = 0.03 ± 0.001; *p* < 0.01 for both). In contrast, total Ca^2+^ retention after an equilibration margin of 200 s was most pronounced in IR15/30 compared to all other groups (p < 0.01 vs. IR0/30, *p* = 0.01 vs. IR30/30, p < 0.01 vs. IR40/30). Between the remaining groups, no significant difference could be detected.

**Fig. 5:**
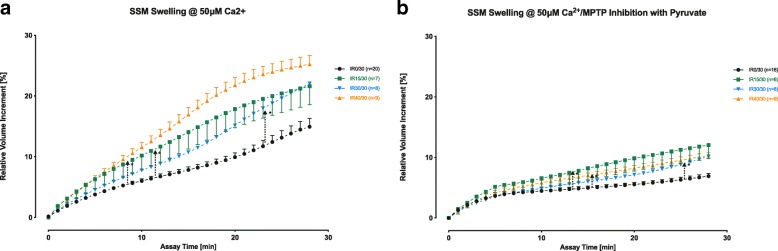
Calcium-induced swelling of SSM. Ca^2+^-induced swelling of SSM without (**a**) and with (**b**) pyruvate-dependent blocking of mPTP. Arrows indicate the earliest pair (IR/30 vs. IR15/30, IR 30/30, IR 40/30) of significantly different observations during reperfusion. Values are presented as means ± standard error of the mean; *p ≤ 0.05. SSM: subsarcolemmal mitochondria; mPTP: mitochondrial permeability transition pore

Ca^2+^ sensitivity of mPTP was determined from Ca^2+^-induced SSM swelling (Fig. 5). Compared to IR0/30, excess swelling of all SSM exposed to previous ischemia reached statistical significance, albeit at different time points (IR 40/30: 8 min, IR30/30: 23 min, IR15/30: 11.5 min). Inhibition of mPTP opening with pyruvate caused a marked reduction of the area under the curve (AUC) of mitochondrial swelling in all experimental groups (AUC normalized to IR0/30 without pyruvate, values with vs. without pyruvate: IR0/30: 1.00 vs. 0.61, IR15/30: 1.68 vs. 1.00, IR30/30: 1.42 vs. 0.75, IR40/30: 1.97 vs. 0.85, *p* < 0.01 each). Furthermore, the time to reach significance in relation to IR0/30 was delayed for all ischemia groups (IR 40/30: 16 min, IR 30/30: 24.5 min, IR15/30 12.5 min).

## Discussion

We focused on the integrity and function of subsarcolemmal mitochondria (SSM) after exposure of isolated rat hearts to a clinically relevant spectrum of warm global ischemia ranging from 0 to 40 min followed by a sufficiently equilibrating 30 min of reperfusion. For example, such circumstances might be induced during warm ischemia time in heart transplantation or by insufficient administration of cardioplegic solution in the hypertrophied heart. We chose a Langendorff perfusion model at a constant 37 °C to closely mimic the ischemic conditions in cardiac surgery patients on normothermic extracorporeal circulation. From a clinical point of view, other models like in vitro oxygen deprivation of isolated mitochondria would probably oversimplify and exclude any intracellular mitochondrial affection during the ischemic phase.

Ischemia suppresses all complexes of the electron transport chain (ETC) to a certain extent, albeit depending on the time of exposure. Though NADH dehydrogenase seems to be generally more susceptible to IR than succinate dehydrogenase and complex III [10], Venditti et al. found a significant depression of complex II after 20 min of ischemia compared to 45 min for complex I, each regardless of a succeeding 25 min of reperfusion [8]. We detected a significant drop of complex I and II activities in SSM after prolonged ischemic periods > 30 min. Conflicting results have been reported on the impairment of complex I in the course of IR. Hardy et al. reported reperfusion itself to be responsible for reduced complex I activity [11]. Others observed an initial increase of complex I capacity of up to 40% after 10 min of ischemia with a subsequent decline after 20–30 min followed by 5 min of reperfusion [12]. Upon energizing mitochondria at complex I, we too recorded a significant overshoot in O_2_ consumption at IR15/30 during state 3 respiration and a decrease at IR30/30 and IR 40/30. This could indicate an increase in matrix ADP/Ca^2+^ levels in non-lethally damaged SSM. Regardless of a substantially longer reperfusion period, our findings seem to corroborate a model of predominantly ischemic impact on complex I for SSM.

The ETC is the driving force of maintaining a stable, inwardly negative potential (ΔΨm) over the inner mitochondrial membrane (IMM). As physiological mitochondrial Ca^2+^ uptake is largely conveyed by an electrophoretic uniport, ΔΨm restoration during reperfusion induces a substantial rise of mitochondrial calcium levels [13]. We found a distinct hyperpolarization of ΔΨm after complex II activation in IR30/30 and IR40/30 compared to IR0/30 and IR15/30. ΔΨm rapidly dropped to control values after CCCP-induced breakdown of the transmembraneous proton gradient indicating a profound instability of IMM potential in IR30/30 and IR40/30. Both these findings concur with the observed calcium retention kinetics. IMM hyperpolarization led to an initial slowdown of Ca^2+^ release. However, overall mitochondrial Ca^2+^ retention was not influenced due to an impairment of ΔΨm stability over time and the consecutive Ca^2+^ loss.

As complex II-IV activity was reduced during state 3 respiration after prolonged ischemia, ΔΨm hyperpolarization before ETC uncoupling might be explained by ischemia-triggered inhibition of respiratory control. IR15/30 did not alter initial ΔΨm upon activation of complex II suggesting an intact respiratory control mechanism. Short ischemia exerted some stabilizing effect on ΔΨm over time after uncoupling thereby supporting the assumption of rapid recovery from short ischemic periods [14, 15]. Moreover, Ca^2+^ net retention at the end of the uptake/release cycle was also exclusively elevated in IR15/30 as compared to IR30/30 and IR40/30 vs. controls suggesting a causal link towards ΔΨm preservation. This seems to contradict previous findings of Racay et al. who noticed a reduction of Ca^2+^ retention after 15 min of rat brain ischemia [16]. It needs to be pointed out, that this group also reported a progressive inhibition of complex I after ischemia whereas we saw an overshoot in state 3 respiration of SSM exposed to IR15/30 and energized at complex I. While ΔΨm stabilization could not be attributed to hyperactive state 3 respiration in complex I-V in our experiments due to exclusive activation of complex II in ΔΨm investigations, elevated complex I activity might contribute to a transient Ca^2+^ storage within the mitochondrial matrix via ΔΨm surplus charge.

ΔΨm breakdown can be delayed by blocking of the mitochondrial permeability transition pore (mPTP) with cyclosporine A in mitochondria of rat hearts subjected to > 35 min of ischemia [17]. The excessive swelling observed in IR groups might be a result of ΔΨm-hyperpolarization and instability reported above. It has been well described that ΔΨm-hyperpolarization acts as a strong promoter of mPTP-opening. As could be expected from previous studies, IR40/30 was the first group on the timeline to significantly differ from IR0/30. Pyruvate-mediated acidification of the medium blocked mPTP opening with a significant reduction of all individual AUCs. However, the time to reach a significant difference from IR0/30 controls doubled in IR40/30, while it was merely unaffected in both IR15/30 and IR30/30. This emphasizes the importance of mPTP opening after prolonged ischemia in IR pathology of SSM. Here our data clearly demonstrate that mild IR resulting from limited ischemia might be controllable by reperfusion strategies while extended ischemia tends to result in fatal SSM-depolarization.

## Conclusions

We examined a variety of effects of ischemia reperfusion injury on subsarcolemmal mitochondria. Our results resemble those discovered by other groups that were dealing with IR influence on mitochondria in general. Though we did not directly compare our findings to a control group of interfibrillar mitochondria, some distinct SSM properties could be delineated. On the one hand, longer periods of ischemia ≥30 min dealt an expected amount of damage with respect to ΔΨm instability, Ca^2+^-induced swelling and electron transport chain impairment. On the other hand, short ischemic intervals of 15 min seemed to exert some stabilization effect regarding interference with CCCP-mediated disruption of ΔΨm and consecutively increased Ca^2+^ retention capacity. Taken together, these findings suggest important differential effects of ischemia time on functional SSM properties. These effects should be accounted for in any experimental design investigating mitochondrial IR injury as well as possible protective interventions.

Further studies should focus on I) examining the transition point from reversible to irreversible IR injury and II) relating our results to the effects of short periods of ischemia on interfibrillar mitochondria.

## Abbreviations

ADP: Adenosine Diphosphate
ANOVA: Analysis of Variance
ATP: Adenosine Triphosphate
AUC: Area Under the Curve
CCCP: Carbonyl Cyanide m-Chlorophenyl Hydrazine
EGTA: Ethylene Glycol-bis(β-Aminoethyl Ether)-N,N,N′,N′-Tetraacetic Acid
ETC: Electron Transport Chain
Fisher’s LSD: Fisher’s Least Significant Difference
Hepes: 4-(2-Hydroxyethyl)-1-Piperazineethanesulfonic Acid
IFM: Interfibrillar Mitochondria
IMM: Inner Mitochondrial Membrane
IR: Ischemia Reperfusion
JC 1: 5,50,6,60-Tetrachloro-1,10,3,30-Tetraethylbenzimidazolylcarbocyanine Iodide
KHB: Krebs-Henseleit Bicarbonate
LV: Left Ventricle/Ventricular
LVdp/dtmax: Left Ventricular Contractility
LVEDP: Left Ventricular End Diastolic Pressure
LVP: Left Ventricular Pressure
MOPS: 3-(N-Morpholino) Propanesulfonic Acid
mPTP: Mitochondrial Permeability Transition Pore
RC: Respiratory Chain
SD: Standard Deviation
SEM: Standard Error of the Mean
SSM: Subsarcolemmal Mitochondria
ΔΨm: Mitochondrial Membrane Potential
